# The 46, XX Ovotesticular Disorder of Sex Development With Xq27.1q27.2 Duplication Involving the *SOX3* Gene: A Rare Case Report and Literature Review

**DOI:** 10.3389/fped.2021.682846

**Published:** 2021-06-11

**Authors:** Jianlong Zhuang, Chunnuan Chen, Jia Li, Yuying Jiang, Junyu Wang, Yuanbai Wang, Shuhong Zeng, Yiming Lin, Yingjun Xie

**Affiliations:** ^1^Prenatal Diagnosis Center, Quanzhou Women's and Children's Hospital, Quanzhou, China; ^2^Department of Neurology, The Second Affiliated Hospital of Fujian Medical University, Quanzhou, China; ^3^Beijing Genomics Institute-Genomics, Beijing Genomics Institute-Shenzhen, Shenzhen, China; ^4^Neonatal Disease Screening Center of Quanzhou, Quanzhou Women's and Children's Hospital, Quanzhou, China; ^5^Department of Obstetrics and Gynecology, Key Laboratory for Major Obstetric Diseases of Guangdong Province, The Third Affiliated Hospital of Guangzhou Medical University, Guangzhou, China; ^6^Key Laboratory of Reproduction and Genetics of Guangdong Higher Education Institutes, The Third Affiliated Hospital of Guangzhou Medical University, Guangzhou, China

**Keywords:** disorder of sex development, ovotestis, Xq27.1q27.2 duplication, *SOX3* gene, chromosomal microarray analysis

## Abstract

**Background:** Very few reports are available on human XX ovotesticular disorder of sex development involving *SOX3* gene duplication. Here we aim to present a rare case of *SOX3* gene duplication in a person from the Chinese population who exhibits XX ovotesticular disorder of sex development.

**Case Presentation:** A 7-year-old Chinese individual from Fujian province in Southeast China was recruited. The patient presented 46, XX karyotype, absence of sex-determining region Y, and was diagnosed with XX ovotesticular disorder of sex development. Furthermore, SNP array analysis demonstrated that the patient had a 2.2-Mb duplication in the Xq27.1q27.2 region (arr[hg19]Xq27.1q27.2:139,499,778-141,777,782) involving the *SOX3* gene. Additionally, no *SOX3* duplication was observed in the parents or the sibling, who displayed none of the clinical features.

**Conclusion:** We identified the first case of *SOX3* duplication in a Chinese individual who exhibits ovotesticular disorder of sex development. Our study strengthens the link between the *SOX3* duplication and XX ovotesticular disorder of sex development and indicates that *SOX3* is the evolutionary antecedent of sex-determining region Y.

## Introduction

Sex-determining region Y (*SRY*) is the key gene in 46, XY normal males. *SRY* initiates a complex genetic cascade, promoting the differentiation of the testis. However, the coexistence of ovarian and testicular tissues is present in some 46, XX individuals, which refers to as ovotesticular disorder of sex development (OT-DSD) (1, 2). Studies have shown that the occurrence of 46, XX OT-DSD is related to the dislocation recombination on the X and Y chromosomes during the meiosis of the paternal chromosome, which transfers the *SRY* gene from the Y chromosome to X (3), but only few patients with 46, XX OT-DSD have a detectable *SRY* gene; most of the subjects show an absence of the *SRY* gene (4, 5). However, the *SRY* gene is present in most of (~80%) 46, XX testicular DSD cases (6). Currently, it is believed that sex determination and differentiation are processes of orderly and coordinated expression of autosomal and sex chromosomes, but with the *SRY* gene, abnormalities in any process can lead to sex abnormalities.

As we know, *SRY* up-regulates the expression of SRY-Box transcription factor 9 (*SOX9*) in bipotential gonads, leading to the differentiation of testicular cells and eventually testicular differentiation (7). Moreover, a study has shown that ectopic *SOX9* expression induces the formation of mouse testis in XX gonads (8). Recently, several cases have been reported to carry *SOX9* duplications (9–12), which have been proposed to be responsible for *SOX9* expression during gonad development. SRY-Box transcription factor 3 (*SOX3*), located on the chromosome X (Xq27.1), is a member of the SRY-Box transcription factor family (13). Duplications involving the *SOX3* gene have been reported to be associated with developmental delay, intellectual disability, growth hormone deficiency, infundibular hypoplasia and hypopituitarism, etc. (14, 15).

Recently, XX sex reversal has been reported in transgenic mice with ectopic *SOX3* expression and observed in 46, XX DSD patients with duplications of *SOX3* or genomic rearrangements within the *SOX3* regulatory region (16). Few reports are available on 46, XX *SRY*-negative males with *SOX3* duplications, though a recent study conducted by Tasic et al. revealed a 46, XX male who presented congenital anomalies of kidneys and the urinary tract and had a duplication on chromosome Xq27 involving the *SOX3* gene, indicating links between *SOX3* gene dosage and kidney malformations and sex determination (17). Moreover, a study has shown a 46, XX *SRY*-negative individual with duplication of the *SOX3* gene exhibiting XX OT-DSD (18). In the present study, we describe a 7-year-old OT-DSD case with Xq27.1q27.2 duplication involving the *SOX3* gene, which was first identified in Chinese individuals and additionally strengthened the pathogenic role of *SOX3* duplication in XX OT-DSD.

## Case Presentation

The patient comes from Quanzhou City, Fujian province, in Southeast China. The child was delivered vaginally with a birth weight of 3.7 kg. There was no family history of DSD, and the parents denied any consanguinity. A physical examination showed the child presented ambiguous sex, coronal hypospadias, a penis or enlarged clitoris, and the presence of a scrotum but non-palpable gonads. Subsequent ultrasonography indicated that the patient might have the coexistence of testicular and ovarian tissues on the left side and testicular tissue on the right side. After clinical consultation, the family decided to raise the child as a male, and ovariectomy was performed at 10 months after birth, to remove the ovarian section from the left ovotesticular area. The subsequent histology analysis confirmed the presence of unilateral ovotestes tissues in the left side of the patient.

At age 7, the child's height (130 cm) and weight (23 kg) were within the normal ranges. Hormonal laboratory tests showed low luteinizing hormone (<0.20 mIU/ml), follicle-stimulating hormone (1.21 mIU/ml) and testosterone (<0.10 ng/ml). Serum progesterone and prolactin were normal. Currently, human menopausal gonadotropin (menotropins for injection, AnHui BBCA Pharmaceutical Co., Ltd.) is injected for treatment with 150 U a day. Regular follow-up showed normal penile and testicular development, with normal morning erection.

**Figure 1:**
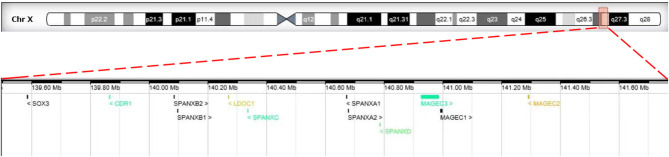
The breakpoint of Xq27.1q27.2 duplication in our study. The upper panel shows the schematic representation of chromosome X. The red rectangle displays the genomic location of Xq27.1q27.2 on the chromosome X. The lower panel presents a magnified view of the Xq27.1q27.2 duplication (arr[hg19] chrX: 139,499,778- 141,777,782) involving the *SOX3* gene.

Chromosome G-banding analysis revealed a normal karyotype (46, XX) in the patient. The parental karyotypes were normal as well. Chromosomal microarray analysis demonstrated that the patient had a 2.2-Mb duplication in the Xq27.1q27.2 region (arr[hg19]Xq27.1q27.2:139,499,778-141,777,782) of the X chromosome (Figure 1). The duplication contains 12 Online Mendelian Inheritance in Man (OMIM) genes: *CDR1, LDOC1, MAGEC1, MAGEC2, MAGEC3, SOX3, SPANXA1, SPANXA2, SPANXB1, SPANXB2, SPANXC*, and *SPANXD*. SNP array analysis was also performed on parental and sibling blood samples. Chromosomal microarray results showed that none of the Y chromosome was observed, and further study indicated that no *SRY* gene was observed in the patient by polymerase chain reaction. Furthermore, no *SOX3* duplication was observed in the parents or the sibling with normal phenotype.

## Discussion and Conclusion

OT-DSD is the disease defined as presence of both male and female gonads. The *SRY* gene is present in few cases of 46, XX OT-DSD patients, which can explain testicular development (18). However, the *SRY* gene is absent in the most of 46, XX OT-DSD patients, and the mechanism underlying the testis development is not fully understood.

*SOX* genes are considered key players in the regulation of nervous system development and embryogenesis; they encode transcription factors that act as key regulators in a variety of developmental processes, including specification, gastrulation, cellular differentiation, and neural induction (19). *SOX9* is critical to the human testis differentiation, while it is still poorly understood whether *SOX3* expression affects sex differentiation. A study showed knockout of *SOX3* did not cause any defects of sex determination; however, affected testis differentiation and oocyte development were observed in *SOX3*-null mice (20). Moreover, another study showed that *SOX3* mutations were absent in the subjects diagnosed with 46, XY gonadal dysgenesis and 46, XX sex reversal, indicating that *SOX3* might not be involved in testis differentiation (21). Recent studies have shown that several human XX male sex reversal cases present rearrangements of the *SOX3* locus, suggesting that a defect in the *SOX3* gene might result in XX male sex reversal in mice and humans. Therefore, researchers believe that *SRY* may arise from *SOX3* and the two genes have interchangeable functions in sex determination (16, 22).

The study conducted by Sutton et al. (16) showed three patients with XX male sex reversal exhibiting rearrangements encompassing or in proximity of *SOX3*. Patient A had two microduplications, one of which covered the entire *SOX3* gene; patient B carried a microdeletion located upstream of *SOX3* in Xq27.1; patient C had a large duplication that encompassed the *SOX3* gene and at least 18 additional genes, which might be responsible for the clinical phenotype (Table 1). Additionally, another study by Moalem et al. (22) showed *de novo SOX3* gene duplication in XX male sex reversal with genital abnormalities. The patient exhibited a partial sex reversal with abnormal genitalia and had three copy number variants, the first of which was a 494-kb duplication in region Xq27.1, which encompassed the *SOX3* gene. The phenotype might be associated with weak or slightly late ectopic expression of *SOX3* in the early gonads. Subsequently, a study (18) identified the first 46, XX OT-DSD case who showed a *SOX3* duplication, with absence of *SRY*, and presented hypospadias and bilateral cryptorchidism (Table 1).

**Table 1:** Literature review of the involvement of *SOX3* duplications in disorders of sex development.

	**Wood et al., Family A Patient 1**	**Wood et al., Family APatient 2**	**Sutton et al., Patient A**	**Sutton et al.,Patient C**	**Moalem et al., Patient 1**	**Grinsponet al., Patient**	**Our case**
Disorders of sex development	XX male reversal.	XX male reversal.	XX male reversal.	XX male reversal.	XX male reversal.	OT-DSD	OT-DSD
Age	7 years	2.5 years	30 years	1.5 years	1 year	2.5 years	7 years
Growth and developmental issues	GH deficiency; Normal serum prolactin and cortisol. Psychomotor development.	History of neonatal hypoglycemia; severe cortisol, TSH, GH, and gonadotrophin deficiency. He has normal psychomotor development but has been noted to be hyperactive.	Normal	Developmental and growth delay; microcephaly.	Normal	Normal	Normal
Genitals and testis	Details not reported.	Hypoplastic genitalia, with both testes palpable high in the inguinal canal and a micropenis.	Details not reported.	Right testicles appear smaller than left; Hypoplastic scrotum; testes are retractile and can be brought down.	Penoscrotal hypospadias with a bifid scrotum; phallus was otherwise unremarkable with erectile tissue palpable; on ultrasound epididymis appearing grossly normal.	The testicular tissue and ovarian tissue all exist.	The ovotesticular tissue on the left side and the testicular tissue on the right side.
Vulva	Male	Male	Male	Male	Bilateral cryptorchidism.	Hypospadias and bilateral cryptorchidism.	Hypospadias and bilateral cryptorchidism.
SRY	Unknown	Unknown	Negative	Negative	Negative	Negative	Negative
Genotypes	Tandem duplication 685.6 kb in length on the X chromosome that spanned the *SOX3* gene.	Tandem duplication 685.6 kb in length on the X-chromosome which spanned the *SOX3* gene	Two microduplications were observed, the first of which spanned the entire *SOX3* gene	6 Mb duplication that encompasses *SOX3* and at least 18 additional distally located genes.	Three microduplications were observed, the first of which contains the *SOX3* gene.	502 kb duplication that encompasses *SOX3* gene and its regulatory sequences.	2.2 Mb duplication that encompasses *SOX3* gene.
Inheritance	Maternally derived.	Maternally derived	Unknown	Unknown	*De novo*	*De novo*	*De novo*

In our study, we secondly identified a 46, XX OT-DSD case with *SOX3* gene duplication, which was first found in Chinese individuals. The findings are in agreement with the previous studies, supporting the notion that duplication of *SOX3* is responsible for partial testicular differentiation in the fetal XX gonads. In this study, apart from the *SOX3* gene, 11 OMIM genes were also involved in this duplicated region. Previous studies indicated that the *MAGE* genes and *SPANX* genes are specifically expressed in tumors and testis (23–25), which needs further investigation as to the potential relationship between these genes and sex determination.

In conclusion, our study firstly identified a patient carrying the Xq27.1q27.2 duplication involving the *SOX3* gene in a 46, XX OT-DSD Chinese individual, which provides additional evidence that the duplication of *SOX3* is pathological in the XX OT-DSD and further indicates that *SOX3* may be the evolutionary antecedent of *SRY*. However, more work can be done on the expression of *SOX9* or other genes that affect the gonadal differentiation pathway, such as *WNT4* or *RSPO1*.

## Data Availability Statement

The raw data supporting the conclusions of this article will be made available by the authors, without undue reservation.

## Ethics Statement

The studies involving human participants were reviewed and approved by Ethics Committee of Quanzhou women's and children's hospital. We confirmed that all subjects who participated in this study signed written informed consent for publishing their own and their children's genetic data and relevant information.

## Author Contributions

JZ and CC designed the study and wrote the article. JW, SZ, and YW performed the karyotype analysis and analyzed the data. YJ, JL, YL, and YX revised and polished the manuscript. All authors approved the final article.

## Conflict of Interest

The authors declare that the research was conducted in the absence of any commercial or financial relationships that could be construed as a potential conflict of interest.
